# Rapid and Reversible Recruitment of Early Visual Cortex for Touch

**DOI:** 10.1371/journal.pone.0003046

**Published:** 2008-08-27

**Authors:** Lotfi B. Merabet, Roy Hamilton, Gottfried Schlaug, Jascha D. Swisher, Elaine T. Kiriakopoulos, Naomi B. Pitskel, Thomas Kauffman, Alvaro Pascual-Leone

**Affiliations:** Department of Neurology, Berenson-Allen Center for Noninvasive Brain Stimulation, Beth Israel Deaconess Medical Center and Harvard Medical School, Boston, Massachusetts, United States of America; Istituto di Neurofisiologia, Italy

## Abstract

**Background:**

The loss of vision has been associated with enhanced performance in non-visual tasks such as tactile discrimination and sound localization. Current evidence suggests that these functional gains are linked to the recruitment of the occipital visual cortex for non-visual processing, but the neurophysiological mechanisms underlying these crossmodal changes remain uncertain. One possible explanation is that visual deprivation is associated with an unmasking of non-visual input into visual cortex.

**Methodology/Principal Findings:**

We investigated the effect of sudden, complete and prolonged visual deprivation (five days) in normally sighted adult individuals while they were immersed in an intensive tactile training program. Following the five-day period, blindfolded subjects performed better on a Braille character discrimination task. In the blindfold group, serial fMRI scans revealed an increase in BOLD signal within the occipital cortex in response to tactile stimulation after five days of complete visual deprivation. This increase in signal was no longer present 24 hours after blindfold removal. Finally, reversible disruption of occipital cortex function on the fifth day (by repetitive transcranial magnetic stimulation; rTMS) impaired Braille character recognition ability in the blindfold group but not in non-blindfolded controls. This disruptive effect was no longer evident once the blindfold had been removed for 24 hours.

**Conclusions/Significance:**

Overall, our findings suggest that sudden and complete visual deprivation in normally sighted individuals can lead to profound, but rapidly reversible, neuroplastic changes by which the occipital cortex becomes engaged in processing of non-visual information. The speed and dynamic nature of the observed changes suggests that normally inhibited or masked functions in the sighted are revealed by visual loss. The unmasking of pre-existing connections and shifts in connectivity represent rapid, early plastic changes, which presumably can lead, if sustained and reinforced, to slower developing, but more permanent structural changes, such as the establishment of new neural connections in the blind.

## Introduction

A growing body of evidence suggests that in blind subjects, the visually deprived occipital cortex becomes engaged in crossmodal processing of tactile and auditory sensory input as well as in certain linguistic and verbal memory tasks (e.g. [1]–[6], for review see [7]–[10]). Within the tactile domain, numerous neuroimaging studies have demonstrated activation of occipital cortical areas in blind subjects performing a variety of tactile tasks such as Braille character recognition, discrimination of vibrotactile stimuli, and haptic object exploration [5], [11]–[18]. Behavioral changes in tactile sensory perception following long-term visual deprivation have also been reported. For example, blind Braille readers have been shown to possess superior tactile acuity in their Braille reading fingers compared to other fingers of the hand or those of sighted control subjects [19]–[23].

In the blind, the functional relevance of occipital cortex in tactile processing is highlighted by a case-study reporting the sudden onset of Braille alexia in a congenitally blind proficient Braille reader following acute bilateral occipital cortical damage [24]. The functional role of occipital cortex has also been investigated by transcranial magnetic stimulation (TMS). This technique affords the possibility to examine the effect of transient and localized disruptions of cortical activity on a given perceptual or behavioral task [25]. Cohen and colleagues (1997) and more recently, Kupers and coworkers (2007) have used repetitive transcranial magnetic stimulation (rTMS) delivered to occipital cortex to disrupt Braille character discrimination abilities in the blind, thereby establishing a causal relationship between tactile processing and cortical function [26], [27].

A critical question in the study of crossmodal processing is whether the recruitment of occipital cortex occurs through changes of existing neural networks or the formation of new neural connections [28], [29]. While these possibilities are neither mutually exclusive nor exhaustive, the functional recruitment of occipital cortex after a short period of visual deprivation (i.e. days) in an adult brain would suggest that an unmasking of existing connections (as opposed to the growth of new axonal projections) may underlie these changes. Anatomical support demonstrating the existence of long-range cortico-cortical connections between early sensory cortices in non-human primates has been reported [30]–[33]. In a recent study, Wittenberg and colleagues applied rTMS over somatosensory cortex in early blind subjects and found a corresponding increase in regional cerebral blood flow (rCBF) within striate and extrastriate visual areas using positron emission tomography [34]. This activation was also found in late blind individuals, albeit to a lesser degree, but not in sighted controls [34]. However, there is accumulating evidence demonstrating that even in sighted individuals, occipital visual areas are implicated in crossmodal processing during performance of certain tactile tasks [35]–[44].

We have previously shown that normally sighted subjects blindfolded for a period of five days perform better than non blindfolded controls in a Braille character discrimination task (Kauffman et al., 2002). Furthermore, visual deprivation for five days also leads to dramatic changes in the overall excitability of occipital cortex (quantified by phosphene thresholds using TMS [45]). Thus, we hypothesized that if crossmodal recruitment of the occipital visual cortex for tactile processing is related to an unmasking of connections, a prolonged period of sudden and complete visual deprivation might be sufficient to reveal their existence and functional significance. Here, we present the results of a long-term study in which normally sighted subjects underwent sudden and complete visual deprivation for a period of five days while immersed in an intensive Braille instruction and tactile training program. Behavioral performance was investigated at the onset, during and after the blindfold period. In a first experiment we used functional magnetic resonance imaging (fMRI) to characterize hypothesized changes in activity of the occipital visual cortex in response to tactile stimulation during and following the blindfold period. In a second experiment we investigated the functional significance of such changes with rTMS to elucidate the specific causal contribution of the occipital cortex with regards to tactile processing. Different subjects were studied in these experiments to avoid the potential confounding effect of TMS on fMRI BOLD activity.

## Results

**Figure 1:**
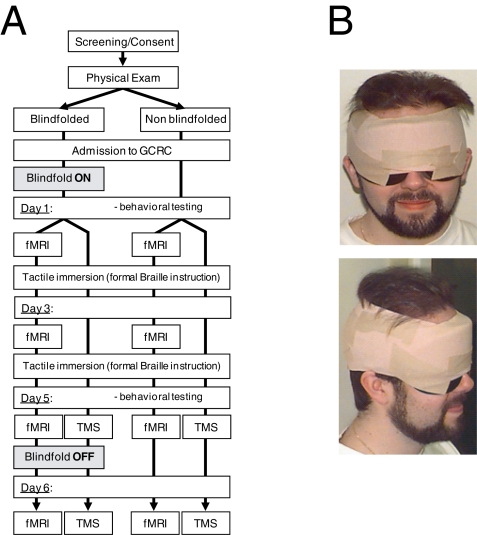
Study design. (a) Subjects were randomized to a blindfolded or non blindfolded group. Tactile behavioral assessments were obtained at day 1 and day 5. Both groups were immersed in an intensive tactile stimulation program that included formal Braille instruction. A subset of subjects participated in a neuroimaging study on day 1, day 3, day 5, and day 6 (i.e. 24 hours after blindfold removal) and a different subset of subjects participated in a TMS study (day 5 and day 6) (b) Photograph of a subject wearing the blindfold used in the study.

A total of 47 normally sighted subjects were enrolled and participated in different components of the study (see Methods and figure 1). In the first experiment 32 subjects underwent serial fMRI's during and following the blindfold period. In the second experiment we studied the impact of transient disruption of the occipital cortex with rTMS on tactile Braille reading in 15 subjects after 5 days of blindfolding and 1 day following removal of the blindfold. In both experiments subjects were randomly assigned to a blindfolded and a non-blindfolded study group, and all subjects (blindfolded and non-blindfolded) were immersed in an intensive tactile stimulation program that included intensive Braille reading instruction (4–6 hours a day) and an additional 2 hours each day playing tactile games (for example, tactile dominos) under supervision. Blindfolded subjects were totally visually deprived for a five-day period using a specially designed blindfold that prevented all light from reaching the eyes while allowing full range of eye motion, including opening and closing of the eyelids. The blindfold was verified every morning and evening to ensure secure positioning, attachment and comfort. During pilot testing, each subject's blindfold had a piece of photographic paper attached to the inside in order to confirm that no light exposure occurred. The blindfold was applied on a Monday (day 1) in the early morning and removed on Friday (day 5) in the late afternoon. Non-blindfolded subjects wore the same type of blindfold for all testing sessions and during the daily tactile stimulation program. Therefore, non-blindfolded subjects were in effects blindfolded for up to 8 hours some days (and for at least 6 hours each day).

### Effect of Blindfolding

All blindfolded subjects were able to tolerate the five-day blindfolding period. Only one subject tampered with (and might have temporarily removed) the blindfold while sleeping and was therefore excluded from further study. Approximately half of the blindfolded subjects complained about itching around the eyes on the second or third day of the blindfold period. For these subjects, the blindfold was removed in a completely dark room to prevent accidental visual exposure to light and replaced with a new one.

As previously reported [46], [47], most blindfolded subjects described spontaneous and complex visual hallucinations after the first or second day of blindfolding. The visual descriptions were reminiscent of those typically described in Charles Bonnet Syndrome and included very vivid images of persons and objects. Interestingly, in most subjects these hallucinations coincided with the observed period of a maximal increase in cortical excitability on day 1 and day 2 [45] and generally subsided over the course of the study. None of the non-blindfolded subjects reported such hallucinations. For a full description of these results see [47].

Following blindfold removal, adjustment to the normal sighted condition occurred without complications in all subjects, as confirmed by ophthalmic examination. Initially, subjects reported feeling disoriented and described visual symptoms of palinopsia (a visual disturbance in which images persist after the stimulus is gone) and impaired depth perception. In general, the adjustment took approximately 30–45 min with the gradual increase of ambient lighting under the subject's own control. No changes in performance in an extensive battery of neuropsychological testing were noted in either group of subjects during or following the blindfolding period.

### Tactile Perception Effects

As part of experiment 1, we examined tactile processing using von Frey hairs, a grating orientation task (GOT), and a Braille character discrimination task, to see if performance levels in the two groups of subjects changed significantly over the course of the five-day study period.

**Figure 2:**
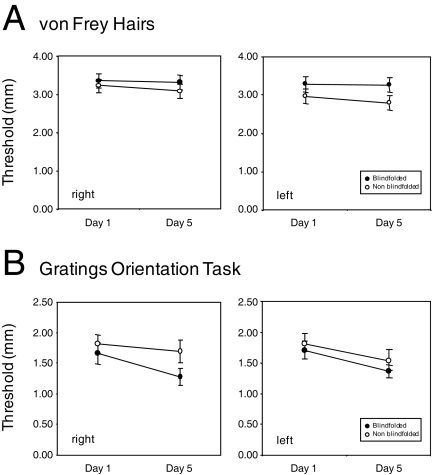
Behavioral results. Group performance assessing touch thresholds with (a) von Frey Hairs and (b) tactile spatial acuity with JVP Domes over the course of the study. Performance is separated by right and left hand (blindfold: filled symbols, non blindfold: open symbols). Overall performance improved over the course of the study period in both the blindfold and blindfold groups.

Analyzing performance on the von Frey hair task (figure 2A) suggested that threshold values had improved significantly in both groups over the course of the five-day period. A repeated measures ANOVA revealed a significant main effect of day (day 1 vs. day 5; F(1,30) = 5.78, p<0.05) and hand (right vs. left; F(1,30) = 8.73, p<0.01). With respect to day, we found thresholds to be lower on day 5 (mean±sem = 3.09 mm±0.04) compared to day 1 (3.24 mm±0.04). Thresholds also appeared to be lower in the left hand (3.10 mm±0.04) compared to the right hand (3.23 mm±0.04). The factor of group (blindfold vs. non blindfold) had no significant effect (p>0.05) suggesting that performance in both study groups was similar over the five-day period. None of the other factors or possible interactions were significant.

Performance on the GOT task (figure 2B) revealed a significant effect of day (F(1,27) = 2.24, p<0.01) suggesting that overall, thresholds decreased over the five day period (compare means of 1.75 mm±0.02 for day 1 and 1.47 mm±0.02 for day 5), while the factors of hand and group did not show any significant effect (each p>0.05). As with the von Frey hairs task, the factor of group (blindfold vs. non blindfold) had no significant effect (p>0.05) again suggesting that performance in both study groups was similar. None of the other possible interactions were significant (p>0.05).

**Figure 3:**
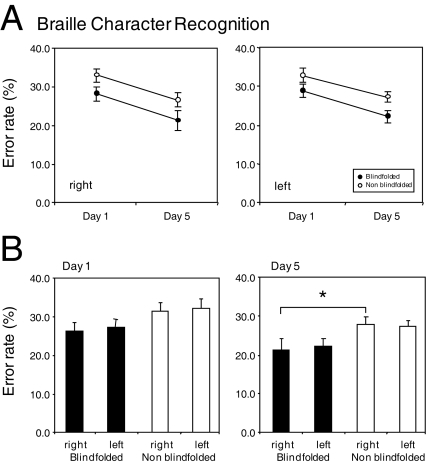
Behavioral results. Group performance on the Braille character discrimination task for both study groups over the course of the study. (a) Performance separated by right and left hand. Note that there is an overall improvement (decrease in error rate) over the five day period in both study groups. (b) Performance separated by day of study. Note that on day 1 of the study, no statistical significance in performance was observed between the groups however performance for the right (trained) hand was significantly better for the blindfold group compared to non blindfold controls after 5 days of blindfolding (* = p<0.05).

For the Braille character discrimination task (figure 3A), complete data for both hands and both testing days (days 1 and 5) was available for only 15 subjects (7 blindfold, 8 non blindfold). A Mann-Whitney test (two-tailed, α = 0.05) revealed a significant effect of group at day 5 (U = 10.0; p<0.05) whereas the two groups were not significantly different at day 1 (p>0.05). Braille character recognition improved in both groups over the five-day study period (see figure 3B). However, the improvement was greater in the blindfold group (compare 24.29%±1.57 for the blindfold group and 29.65%±1.90 for the non blindfold group). Given that all the study subjects were trained to read Braille using only their right hand, we further compared performance in both hands. As there was no *a priori* reason to consider that the influence of handedness would be different between the two groups at baseline (day 1), a t-test was run considering all subjects in both groups. No significant effect of hand was observed on either day 1 (t(14) = 1.15, p>0.05) or day 5 (t(14) = 0.14, p>0.05).

### Neuroimaging

**Figure 4:**
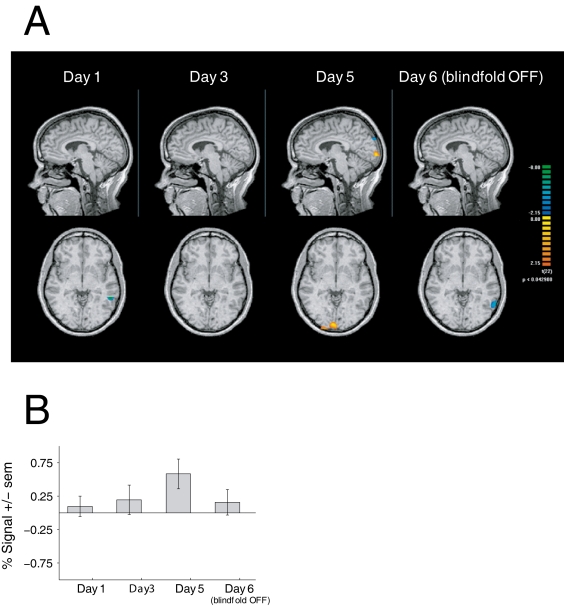
Neuroimaging results. (a) Differential tactile activation maps contrasting the blindfold and non blindfold groups are shown for each fMRI session of the study. (b) A region of interest (ROI) was defined by the area of occipital activation found on day 5 in the group maps. The average differential tactile activity between groups in this ROI is plotted here in z-score units across days of the study. The difference between groups was significant on day 5, as expected from the definition of the ROI, but did not reach significance on any other day.

We hypothesized that functional imaging would show differential tactile activation in occipital cortex between the blindfolded and non-blindfolded groups, and that this activation would increase in magnitude over the week of visual deprivation. In an initial exploratory analysis, we examined tactile activity on day 5, at which time the differential effects of blindfolding were expected to be greatest. This contrast revealed significantly greater tactile activation in the blindfold group than the controls within a bilateral region of occipital cortex, including the calcarine sulcus. This activation was not evident earlier in the week on day 1 or day 3, nor was it found 24 hours after removal of the blindfold on day 6 (figure 4A).

This area of significant differential occipital activity found on day 5 was used to define a region of interest (ROI) for additional confirmatory analysis. For each day's scan data, voxel time series within this ROI were first averaged together within subjects and subsequently analyzed using the same general linear model (GLM) as for the whole-brain voxelwise analysis. The regressor weights resulting from the fit GLM were extracted and entered into an ANOVA with factors “group” (blindfold vs. non-blindfold) and “hand” (right vs. left). Tactile activity in this occipital ROI showed a clear trend across days towards increasing differential activation between the blindfold and non-blindfold control groups, with the greatest difference found at the end of the blindfolding period on day 5 (figure 4B). This difference is nearly abolished after the blindfold was removed on day 6 (figure 4B). The main effect of blindfolding reached significance on day 5 (F(1,22) = 6.92, p<0.05), as expected from the definition of the ROI, but did not approach significance on any other day (all p>0.1). The effect of hand never approached significance (all p>0.1). Several other regions showed greater activation in the non-blindfolded control group on day 5 including the left intraparietal sulcus and insula bilaterally (Table 1).

**Table 1:** Talaraich coordinates of areas of significant activation observed on day 5.

Area	Talaraich Coordinates		
	x	y	z
(Blindfold>Non Blindfold)			
- Occipital	7	−96	−3
(Non Blindfold>Blindfold)			
- Precentral Gyrus	52	−1	44
- Right Insula	37	−8	18
- Left Intraparietal Sulcus	−28	−76	35
- Left Insula	−36	−12	23

### Effect of rTMS Disruption

**Figure 5:**
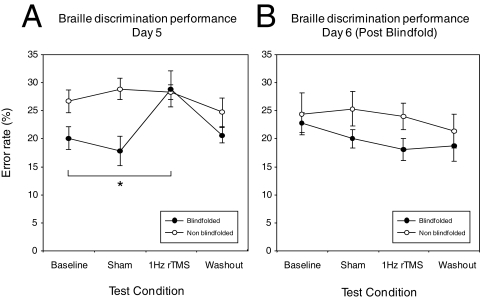
Effect of TMS on Braille discrimination. The effect of 1Hz rTMS on Braille discrimination ability (% error rate) is compared to baseline performance, sham stimulation and after a 10 min washout period. On day 5 (part a), rTMS (but not sham) had a significant effect on Braille character discrimination in the blindfold group (filled symbols) but not in the non blindfolded group (open symbols) (* = <0.05). This effect was not present in either the blindfold or non blindfolded group on Day 6 (part b).

Repetitive TMS was used to transiently disrupt the function of occipital cortex in both the blindfold and non-blindfold subjects. This experiment was completed in a different group of subjects than experiment 1 since rTMS might induce changes in fMRI BOLD activity and could thus have affected the brain imaging results. The effects of rTMS were not tested until the last day of blindfolding (day 5) in order to avoid modifying plastic changes related to the blindfolding. Follow-up testing was conducted 24 hours after blindfold removal (day 6). At day 5 (figure 5A), baseline Braille reading performance was compared to performance following rTMS, sham stimulation, and after a 10 min washout period using a within group non-parametric analysis (Wilcoxon signed ranks test) and error rate as the dependent variable. In the blindfold group, comparing baseline performance (mean error rate±sem = 21.27%±2.99) to sham stimulation (17.78%±2.62) did not reveal a significant effect (p>0.05). However, Braille character recognition performance following rTMS (28.89%±3.22) was significantly worse than baseline performance (p<0.05; 1.99). Comparing performance after the sham stimulation condition and rTMS was also significant (p<0.05; 0.89). No significant effect of TMS was observed comparing baseline performance to washout (20.64%±1.35, p>0.05; 1.28). In the non-blindfold group, no significant effect of rTMS was revealed comparing baseline performance to either sham (p>0.05; 1.28), rTMS (p>0.05; 1.11) or washout (p>0.05; 0.77). Mean error rates (%±sem) for baseline, sham, rTMS and washout were 26.67%±2.38, 28.89%±1.88, 28.33%±1.32, 24.72%±2.54 respectively.

Comparing performance across conditions on day 6 (figure 5B) showed no significant effect of rTMS with respect to baseline performance in either the blindfold or the non-blindfold control group (p>0.05; 1.70; p>0.05; 0.37 respectively). Similarly, no significant effect was observed when baseline performance was compared to the sham and washout conditions in either the blindfold (p>0.05; 0.85, p>0.05;1.47 respectively) or non-blindfold group (p>0.05; 0.54, p>0.05;1.63). Mean error rates (%±sem) for baseline, sham, rTMS, and washout were 22.86%±1.73, 20.80%±1.68, 18.10%±1.97, 18.73%±2.70 for the blindfold group and 24.07%±2.66, 25.33%±2.47, 24.00%±1.88, 21.33%±2.40 for the non blindfold group respectively.

Finally, the effect of blindfold removal was characterized by comparing performance following the washout period on day 5 with performance levels at baseline on day 6. A pair-wise comparison of blindfold and non-blindfold participants revealed no significant difference in performance for either group (p>0.05 for both groups). This finding suggests that Braille character discrimination performance remained stable for both study groups when assessed after the 24-hour blindfold removal period.

## Discussion

The results of this study demonstrate that five days of complete visual deprivation in normally sighted adults combined with intensive tactile training leads to reversible, functional and behaviorally relevant crossmodal changes implicating occipital visual cortex. These results extend our earlier reports and findings [45], [46], [48] and are in line with current evidence implicating the occipital cortex in crossmodal tactile processing.

We found an overall behavioral improvement in tactile perception as determined by von Frey hairs and a grating orientation task (GOT). This effect was equal for blindfolded and non-blindfolded subjects. However, consistent with our results from a previous study [46], tactile Braille character discrimination showed a greater improvement in the blindfold group as compared to the non-blindfold controls. Using serial fMRI, changes in behavioral performance were further characterized by comparing occipital activity in response to tactile stimulation over the five-day study period. On day 5, significantly greater activation was evident within a bilateral region including the calcarine sulcus. This activation was not evident at baseline (day 1), or mid week (day 3), nor was it found 24 hours after removal of the blindfold (day 6). Activation in occipital cortex was also evident on the fifth day in response to either right or left hand stimulation, which is consistent with the behavioral improvement in tactile Braille character discrimination observed with both hands. These results suggest that there is an association between the crossmodal activation seen in occipital cortex and the behavioral effect observed following the five-day visual deprivation period. Support that these crossmodal changes are behaviorally relevant comes from the final rTMS experiment. Specifically, reversible disruption of occipital cortex on day 5 by rTMS impaired Braille character discrimination ability in the blindfold group but not in the non-blindfold controls. No disruptive effect was evident in either group when tested on Day 6 (24 hours after blindfold removal) despite baseline performance being similar to final performance levels at the end of testing at day 5. These findings suggest that the crossmodal changes observed after five days of blindfolding are indeed functionally relevant. The lack of a disruptive effect from applying rTMS 24 hours after the blindfold was removed suggests that the restoration of vision for this period of time was sufficient to rapidly reverse the induced neuroplastic changes.

### Effects on tactile perception

The cerebral networks and neuroplastic mechanisms underlying crossmodal recruitment of visual cortex following visual deprivation remain unclear (see [7] for review). However, the rapidity of the neuroplastic changes described herein supports the view of an “unmasking” of latent multimodal connections underlying the recruitment of occipital cortex for tactile processing [48]. It would seem reasonable that preexisting connections projecting to visual cortical areas may be selectively activated for the crossmodal recruitment of visual cortical areas for non-visual tasks. After prolonged visual deprivation, these same connections may become enhanced through synaptic reinforcement or greater recruitment of contributory neural networks based on task demands. It has also been proposed that top-down signals originating within multimodal associative cortical areas may selectively recruit appropriate primary sensory areas [40]. Changes in connectivity implicating subcortical pathways have also been proposed [49]. Anatomical evidence of direct long-range cortico-cortical pathways connecting primary sensory cortex with other sensory or multimodal cortices has been reported [30]–[32], [50], [51]. In the sighted brain, these same connections could potentially underlie crossmodal tactile processing but remain comparatively suppressed under conditions when vision is present. Under sudden and complete visual deafferentation and specific task demands, activation in visual cortex can also be observed with shortened periods of visual deprivation.

The recruitment of occipital cortical regions might be strongly influenced by experience, behavioral strategy, and task difficulty. The tasks chosen in this study were meant to reflect different aspects of tactile sensory function. For example, von Frey hairs represent a measure of touch threshold while GOT represents a measure of tactile spatial acuity. Braille character discrimination requires the decoding of specific spatial patterns of dots that encode a linguistic meaning. The improvement seen on von Frey and GOT thresholds may be explained, at least in part, by a practice effect occurring in both groups over the five-day period. On the other hand, in Braille character discrimination (arguably a more cognitively and attentionally demanding task [52]); performance improved in both study groups, but a greater improvement was seen in blindfolded participants. Consistent with our previous findings, this suggests that blindfolding itself conferred a behavioral advantage for the tactile Braille character discrimination. Practice-induced changes alone cannot account for the superior performance of the blindfold group. Further support for this notion comes from the fact that both groups received identical training over the study period. In agreement with our previous reports, we found a comparable beneficial effect of tactile learning with both the index finger of the trained right hand as well as that of the untrained left hand independent of blindfold status [46]. These results are in accordance with previous results by Sathian and colleagues who have proposed that interdigital transfer of tactile learning may result from using multiple fingers in activities requiring touch and haptic exploration which in turn, require divided attention between digits [53], [54].

It is possible that complete visual deprivation may promote a reallocation of attention-related resources to non-visual modalities leading to rapid neuroplastic changes [48], [55]. Overall, these findings parallel findings in early blind subjects who show better than normal GOT thresholds [22] but normal performance in the von Frey hair task [56]. Furthermore, this view is in accordance with the notion that advantages in tactile perception encountered in blind subjects are due to the learning of perceptual skills and underlying changes in crossmodal processing rather than increased sensory sensitivity alone [21], [57]. Anecdotal reports from Braille instructors have documented that visual input may be actually *detrimental* to the acquisition of Braille reading skills in subjects recently blind or acquiring tactile Braille reading skills while becoming blind [58]. In fact, partially sighted subjects who learn to read Braille often try to use their residual sight rather than relying solely on touch. This raises the intriguing possibility that blindfolding partially blind subjects may potentially facilitate the Braille learning process [58]. However, it is important to remember that the subjects in this study all had normal vision, were rapidly and completely visually deprived for a fixed period of time and knew that their vision would be restored after the deprivation period–a situation that is markedly different from the case of an individual with progressive visual loss occurring over a protracted period of time. Furthermore, there is an inherent assumption that the underlying neurophysiological mechanisms observed here are the same as what occurs following actual vision loss. We cannot definitively validate this assumption, thus extrapolating our findings to individuals with established visual impairment should be done with caution.

### Neuroimaging findings

Evidence from neuroimaging suggests that occipital visual areas are associated with the observed improved behavioral performance reported in this study. Contrasting blindfolded versus non-blindfolded controls, serial fMRI scans revealed an increase in BOLD signal within the occipital cortex in response to tactile stimulation after five days of visual deprivation. This increase in signal was not present 24 hours after blindfold removal. This suggests that continued tactile stimulation during visual deafferentation parallels an increase in activation within occipital visual areas. The fMRI data results of this study should nonetheless be interpreted with caution. First, no differences in activation were observed when the right and left index fingers were compared even though the subjects were all strongly right-handed and trained to learn Braille using their right index finger. In agreement with the observed behavioral data, it is possible that extensive bimanual haptic experience occurring over the five-day period also led to activation of occipital areas when the non-trained hand was stimulated. Secondly, tactile stimulation in the scanner was provided by using a non-specific and unrelated tactile task (stimulation using a loofa brush). Unlike the actual Braille training tasks for which the study subjects were trained (and later tested with rTMS), subjects in the fMRI study simply remained still and were stimulated passively with the tactile stimulus without performing any explicit behavioral task. This study design issue may also account for the relatively low BOLD signal strength observed as well as the lack of observed difference between stimulated fingers. Finally, no correlation between behavioral performance and degree of signal change within occipital cortex was observed. The demonstration of a positive correlation between behavioral performance and activation would have strongly supported the notion that occipital cortex activation is indeed related to an adaptive behavioral gain (see [1]).

Previous neuroimaging studies have provided evidence that areas within occipital cortex are activated during tactile tasks following visual deprivation [35]–[44]. Studies have also demonstrated activation of occipital cortical areas in normally sighted individuals carrying out complex tactile and haptic tasks [5], [11]–[18]. While retinotopic mapping was not carried out in this study, the activity seen within the calcarine sulcus on day 5 strongly suggests activation of primary visual areas. A more recent study by our group using retinotopic techniques and at higher scanner field strength demonstrated V1 activation while performing a fine spatial tactile discrimination task despite being blindfolded for a period as short as 40–60 minutes [42]. These findings thus support the localization activity seen here.

### TMS results

Convergent support for the functional significance of the crossmodal recruitment of occipital visual areas comes from the rTMS experiment. Reversible disruption of occipital cortex on the fifth day by rTMS impaired tactile Braille character discrimination in the blindfolded group but not in the non-blindfolded controls. It is interesting to note that following the removal of the blindfold on day 6, no differential activation was observed in occipital cortex via fMRI, nor did occipital rTMS cause any disruptive effect on Braille character discrimination. Furthermore, comparing baseline performance to the last performance level on day 5 suggests that the Braille skill was intact despite the 24-hour period of blindfold removal. This latter observation suggests the possibility that while the restoration of vision had returned occipital cortex to its original state, the overall consolidation of the Braille learning task may have occurred within other distal cortical or subcortical areas.

Our results are in accordance with previous studies demonstrating that rTMS delivered to occipital cortical areas can disrupt tactile processing in the blind [26] and in normally sighted subjects if suitable tasks are used [41], [44]. The specific causal link between Braille task and performance within the blindfold group at day 5 speaks against alternative explanations involving other cognitive processes such as shifts in attention resources or visual imagery. Though not systematically tested, there is no *a priori* reason to believe that the visual imagery performance would be different across the blindfold and non-blindfold study groups, across testing session, or different following the removal of the blindfold. Yet, the disruptive effect of rTMS was clearly limited to the blindfold group on the fifth day and not present 24 hours after blindfold removal. These findings suggest that the activation observed after five days of blindfolding is functional in nature. The lack of an rTMS effect after blindfold removal suggests that the restoration of vision is sufficient to rapidly reverse the induced changes to baseline.

It is important to note that the subjects who participated in the fMRI component of the study were not the same as the subjects who underwent rTMS. This was by design in order to avoid the possible confound that TMS itself could induce changes that would be unrelated to task-specific activity measured by fMRI. We have previously reported that the five-day blindfold period leads to dramatic changes in cortical excitability as measured by phosphene thresholds [45]. Thus, it would be difficult to disentangle changes in excitability due to visual deprivation and what could be potentially caused by repeated exposure to TMS. While we cannot ensure definitively that all subjects who underwent fMRI would also show disruptive effects following rTMS, the potential confound created by TMS warrants the separation of the study participants. Finally, it is important to emphasize that the disruptive effect of TMS is occurring within an altered neural network. That is, the effect of TMS disruption, while delivered to occipital cortex, may be exerted at a distance along cortical networks that have undergone functional changes in connectivity due to visual deprivation itself. Such a hypothesis would provide a sensible explanation to the lack of impact of rTMS on tactile Braille character discrimination on day 6, when occipital cortex is no longer significantly activated by tactile stimuli on fMRI, but tactile Braille character discrimination remains better than at baseline, presumably supported by activity at brain regions other than the occipital cortex. Further studies with real-time combination of TMS and functional neuroimaging methodologies may help clarify this issue further.

### Summary

In summary, our findings demonstrate the remarkable ability of the adult human brain to rapidly adapt to the visually deprived state. The relatively rapid crossmodal recruitment of occipital cortex to process tactile information suggests a functional relevance for tactile inputs into visual cortex in sighted subjects. The speed of such crossmodal plasticity suggests an unmasking of pre-existing connections or multimodal capabilities heretofore unsuspected. Perhaps most surprising is the fact that complete visual deprivation appeared to improve overall behavioral performance (as evidenced by a decrease in error rate on the Braille discrimination task) in an activity-dependent manner. Transient blocking of visual cortex process using rTMS at the end of the five days disrupted tactile Braille character discrimination. This effect of rTMS disruption was not observed at baseline nor following removal of the blindfold, nor in the non-blindfolded control group. The causal relation between transient occipital cortex disruption and interference on Braille character discrimination supports the notion that the recruitment of visual cortex in tactile processing is functional in nature and in line with results in studies investigating crossmodal plasticity in the blind.

It remains to be determined whether the recruitment of the occipital cortex for tactile processing in the blind and our blindfolded sighted subjects relies on the same or different neuroplastic mechanisms. This issue notwithstanding, our results do reveal that the potential for the adult brain to reorganize itself is much greater than previously assumed. Furthermore, these findings have important implications in that they contribute to current evidence demonstrating that early sensory areas are not strictly modality specific [48]. We suggest that the superior performance of blindfolded subjects may depend on the activation of occipital visual cortical areas. Fundamental principles underlying neural plasticity in response to visual deprivation may be applicable across other neural systems. Enhanced understanding of the mechanisms of rapid crossmodal plasticity is critical for the fundamental understanding of the adaptation of the human brain to injury and might suggest strategies for neurorehabilitation.

## Methods

### Study Participants

A total of 47 normally sighted participants (aged between 18 and 35 years; 30 females) completed the study and were recruited over a three-year period through announcements in the local academic community. Prior to enrolling in the study, all subjects underwent a physical, ophthalmic and neurological exam and a structured interview to establish their emotional stability and likelihood to withstand the potential stress of being blindfolded for an extended period of time. All subjects were free of any history of neurologic, cognitive, or psychiatric impairments or any central or peripheral disorders of somatic sensation. All participants had 20/20 best corrected vision and were strongly right handed as indicated by the modified Edinburgh Handedness questionnaire [59]. Participants received equal financial compensation regardless of the study group they were randomized to. Written informed consent was obtained from all participants prior to entering the study, which had been approved by the Institutional Review Board of Beth Israel Deaconess Medical Center and the Scientific Advisory Board of the General Clinical Research Center. The study was carried out according to the tenants of the Declaration of Helsinki.

### Overall Study Design

All subjects were enrolled in a six-day stay at the Harvard-Thorndike General Clinical Research Center (GCRC) for safety and continuous monitoring. During this time, subjects participated in a variety of psychophysical and neurophysiological testing batteries performed serially. Subjects were randomized into a blindfolded or non-blindfolded group (figure 1A). Blindfolded subjects were totally visually deprived for a five-day period using a specially designed blindfold (Mindfold Inc., Tucson, AZ). The blindfold prevented all light from reaching the eyes while allowing full range of motion, including opening and closing of the eyelids (figure 1B). Elastic Ace bandages were wrapped around the subject's head and over the blindfold to secure it further and prevent accidental displacement. The blindfold was verified every morning and evening to ensure secure positioning, attachment and comfort. During pilot testing, each subject's blindfold had a piece of photographic paper attached to the inside in order to confirm that no light exposure occurred. The blindfold was applied on a Monday (day 1) in the early morning and removed on Friday (day 5) in the late afternoon. In some cases where the blindfold became uncomfortable, the option was provided to exchange it in a dark room with a new one.

Both groups of subjects (blindfolded and non-blindfolded) were immersed in an intensive tactile stimulation program that included intensive Braille reading instruction (4–6 hours a day) taught by a professional Braille instructor (Carroll Center for the Blind; Newton, MA) using the standard Braille series textbooks from the American Printing House for the Blind. Subjects also spent an additional 2 hours each day playing tactile games (for example, tactile dominos) under supervision and were also taught how to walk with a cane by a professional orientation and mobility instructor (Carroll Center for the Blind; Newton, MA). Subjects were instructed to use their right index finger for Braille reading and were encouraged to use both their hands for all other activities of daily living including washing, eating and exploring their environment. Contrary to the common practice in teaching adventitiously blind subjects, the instructors avoided all advice regarding the use of visual imagery during the classes.

### Tactile Sensory Assessment and Behavioral Studies

Thirty-two subjects (16 blindfolded, 16 non-blindfolded) underwent a series of tactile sensory and acuity threshold determinations. Testing was carried out on day 1 and day 5 in both the blindfolded and non-blindfolded groups.

A touch threshold for the index finger was determined with a set of nine von Frey hairs (Stoelting Co., Wood Dale, IL) (for complete details on this sensory testing method see [56], [60]. The hairs were made of nylon and calibrated to provide the following forces when applied to the skin: 0.1, 0.5,1,2,5, 10, 20, 50 and 100 mN. The skin of the finger pad was touched with the hair roughly perpendicular to the skin surface, and the hair was moved 5 mm after it made contact with the skin. Each hair was applied to the right and left index finger pads, 10 times each. The order of application of the different hairs was random and subjects were unaware of which hair was being applied. Subjects were asked to verbally report if they felt a touch with the hair or not.

Tactile acuity testing was carried out using a set of Johnson-Van Boven-Phillips (JVP) domes by determining a threshold for grating orientation (GOT task). This task has been previously shown to be a reliable measure of tactile spatial acuity [22]. Briefly, these stimuli consist of a set of eight hemispherical plastic domes with gratings of equal bar and groove widths varying from 0.35 mm to 3.00 mm cut into their surfaces (JVP Domes, Stoelting Co., Wood Dale, IL). Domes were presented in pseudo-random fashion to the distal pads of the index fingers of both hands for blocks of 20 trials. The hand was placed comfortably in the supine position and the finger was immobilized using adhesive tape applied to the nail. Gratings were applied manually by an experimenter with moderate force (resulting in approximately 2 mm of skin displacement). Careful attention was given to inspect visually and reject any trials in which movement between the skin and grating occurred. The orientation for each trial was selected from a random number table. For each trial, the grating was applied perpendicularly to the surface of the distal pad with the bars and grooves aligned in one of two orthogonal directions (i.e., along or perpendicular to the long axis of the finger) and held for about 1.5 seconds. Subjects were required to identify the stimulus orientation (two-alternative forced choice paradigm). The grating orientation threshold was determined by interpolating between groove widths spanning 75% correct responses (unless performance was at 75% correct response rate for a particular grating). Performance at this level is midway between chance and perfect performance and is a standard psychophysical threshold criterion. The initial grating selected for testing was one that was easily resolved by a subject (yielding a high percent correct response level). Thereafter, gratings were presented in a descending groove width order until performance approached chance performance (50% correct responses). The order of finger testing was randomized for each subject.

Full data on the Braille character discrimination task was available in 22 subjects (11 blindfold and 11 non blindfold). The task used a specially designed, computer controlled Braille stimulator (for complete device description see [46], [55]. The stimulator device has six small carbon fiber rods measuring 1 mm in diameter and arranged according to the dimensions of a Braille cell. In the “off” position, the tips of the rods are flush with the surface of the stimulator unit and are pushed upwards using a series of miniature pneumatic valves. When pushed up, the rods indent the skin of a finger pad resting against the stimulator cell by 1.5 mm in accordance with the Braille character standards. Custom software allows the investigator to present pairs of Braille characters. Braille character discrimination ability was tested at baseline (day 1) and day 5 of the experiment. All subjects were blindfolded during the testing sessions. A pair of Braille characters were presented in pseudo-random fashion to the pad of the subject's index finger (right or left) and the subject was instructed to verbally identify whether they were the same or different. At each testing session, subjects completed 36 trials in two blocks of 18 trials. Braille character presentation lasted for 100 ms each with a 2300 ms inter-stimulus interval (ISI) between presentations. During the ISI, all rods were flush with the surface of the stimulator cell and the subject's response was recorded. Subjects were given three practice trials prior to the testing of each finger to ensure that the entire finger pad was centered over the Braille cell of the stimulator. Once satisfied with their finger position, a strip of surgical tape was placed over the most distal phalangeal joint to immobilize the fingertip.

### Serial Functional Neuroimaging and Analysis

All imaging studies were performed on a 1.5 Tesla Siemens Magnetom Vision scanner using a standard quadrature head coil. Subjects (16 blindfolded, 16 non-blindfolded) were positioned supine in the scanner with their head supported to restrict head motion. All subjects were blindfolded during the scanning sessions. Imaging parameters sensitive to blood oxygen level dependant (BOLD) fMRI signal were as follows: TR 3000 ms; TE 64 ms; matrix 128×128; FOV 320 mm; slice thickness 8 mm; 10–12 slices acquired approximately perpendicular to the AC-PC line. Anatomical volumes were acquired in each session using a T1 weighted MPRAGE sequence with 1 mm isotropic resolution. Tactile sensory stimulation was provided using a wooden loofa brush (surface area of 11.5 cm×4 cm) moved forward and backward along the length of the distal pad of either the right or left index finger at a rate of approximately 320 strokes/min covering a distance of 1.5 cm. The fingers were stimulated in 60 second blocks alternating between right hand, left hand, and no stimulus (rest) conditions in randomized order, with a 20 second delay between each block. Subjects performed four blocks of each of the three conditions on each day of scanning. Stimulation was provided by the same experimenter throughout the study. The subjects' hands were kept in a pronated position immobilized by padding and with the fingers spread apart to prevent involuntary stimulation of more than one digit at a time.

Functional data analyses, co-registration, and visualization were carried out using BrainVoyager QX 1.7 software (Brain Innovations, Maastricht, Netherlands). Functional data were preprocessed by applying motion correction, 8 mm FWHM spatial smoothing, and temporal linear trend removal. The functional data for each session were then coregistered with the same-session anatomical volumes, resampled into 3 mm isotropic voxels, and transformed into a common Talairach space. The data were then analyzed at the individual subjects level by a general linear model (GLM) with regressors defined to correspond to the time courses of the task conditions, applied to the z-transformed time series of each voxel. The group analysis contrasted the averaged left and right tactile activation between the blindfold and control groups, producing a random effects map of differential tactile activity. These group maps were corrected for multiple comparisons by a joint voxelwise significance/cluster size threshold set to allow detection of weak but spatially extensive activations. The cluster significance level of p<0.05 was estimated through Monte Carlo simulation [61] using the BrainVoyager Cluster Threshold plugin. Group activation maps are shown overlaid on the anatomy of a single representative subject.

### TMS Experiment

This experiment was completed by 15 subjects (7 blindfolded, 8 non blindfolded). Given that delivery of TMS is known to alter cortical excitability [25], the participants in this component of the study (experiment 2) were not the same as those participating in the fMRI experiment (experiment 1). This was by design in order to avoid possible confounds of inducing changes in cortical excitability that potentially could influence the degree of activation revealed by fMRI. All stimulation was delivered using a Magstim 70 mm figure of eight coil with pulses generated by a Magstim Rapid Stimulator (Magstim Inc., Dyfed, U.K., maximum stimulator output of 1.5 T). The stimulation coil was held tangentially, flat against the scalp at the occipital pole (roughly corresponding to the Oz position of the 10-20 EEG reference system). A three-dimensional reconstruction of each subject's anatomical MRI scan was used to determine and maintain the optimal coil placement to target the occipital pole using a frameless stereotaxic neuronavigation device (Integra Radionics, Burlington, MA [62]). A single train of 300 pulses of 1 Hz TMS was delivered to the visual cortex at an intensity of 110% of each subject's motor threshold (determined prior to the onset of the experiment) using previous criteria and parameters within current safety guidelines (Wassermann et al. 1999). During real rTMS, the center of the coil was held tangentially to the scalp and symmetrically across the midline of the occipital pole, targeting the tip of the calcarine fissure. During sham rTMS, the coil was lowered by 3 cm and rotated so that the edge of the two wings of the coil rested at 90 deg on the scalp. In this sham rTMS condition, the induced magnetic field did not enter the brain, although the touch on the scalp, the sound of the coil being activated, and the induced muscle twitching are comparable to those in the real rTMS condition. To avoid carry-over effects between conditions, a wash-out period of at least 30 min between tasks was incorporated between rTMS trains. Overall, each subject received 600 stimuli per train, and a total of 2400 stimuli (1200 real and 1200 sham stimuli).

### Behavioral Data Analysis

Statistical analysis was performed using SPSS v13.0 software (SPSS Inc., Chicago, IL). A three-way repeated measures ANOVA (with factors blindfold×hand×day) was used to test whether performance levels of the groups changed significantly over the course of the five day blindfold period. Significance level was set at p<0.05 and no outliers were removed from the analysis. *Post hoc* comparisons were made using pairwise t-tests using Bonferroni correction. For smaller sample size comparisons, non-parametric analysis considering single-group, paired comparisons were used where appropriate.
